# Localized nasal dermatosis in a healthy male

**DOI:** 10.1016/j.jdcr.2025.10.061

**Published:** 2025-11-07

**Authors:** Aarohi Shah, Kevin Gaddis, Brittney Schultz

**Affiliations:** a University of Minnesota Medical School, Minneapolis, Minnesota; b Department of Dermatology, University of Minnesota, Minneapolis, Minnesota

**Keywords:** autoimmune blistering disorder, localized nasal pemphigus, pemphigus, pemphigus foliaceus, tacrolimus

## Case description

**Fig 1:**
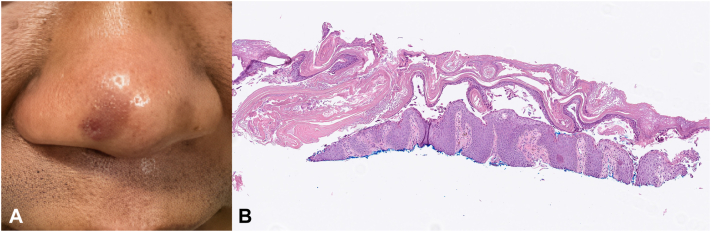
**A,** Hyperpigmented patch on nose with erosion. **B,** Histopathology showing superficial acantholysis. Magnification and stain: hematoxylin and eosin, original magnification ×40.

A 49-year-old male presented to the clinic with a 7 month history of pruritus on his nose. The patient was otherwise healthy and was on no medications. He had no similar eruption elsewhere. He had improvement but not resolution with ketoconazole cream and triamcinolone 0.025% cream. Physical examination revealed hyperpigmented macules with overlying scale on the nasolabial folds and nasal tip with 1 scaly papule (Fig 1, *A*). A biopsy specimen was obtained for histopathologic analysis (Fig 1, *B*).

**Question: What is your diagnosis?**

**A.** Discoid lupus erythematosus
**B.** Pemphigus foliaceus
**C.** Facial discoid dermatosis
**D.** Lupus pernio
**E.** Sarcoidosis

## Discussion

**Fig 2:**
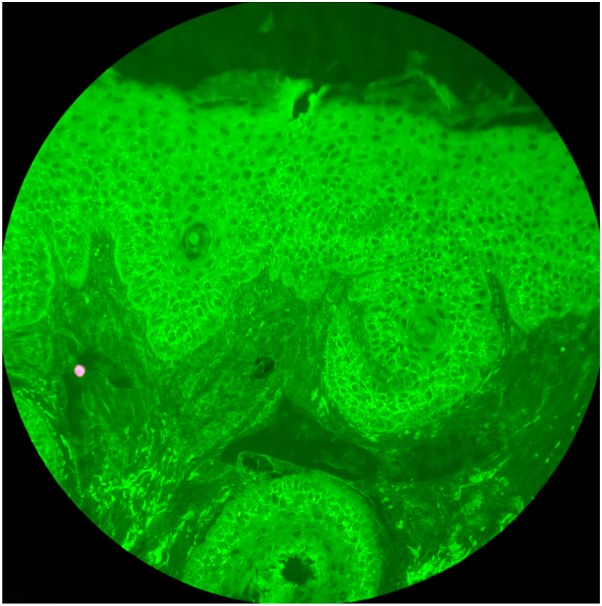
Direct immunofluorescence showing intercellular IgG deposition in lower epidermal keratinocytes. Magnification and stain: direct immunofluorescence—IgG, original magnification ×200.

Histopathologic examination revealed superficial epidermal acantholysis. Direct immunofluorescence (DIF) revealed intercellular immunoglobulin (Ig) G and complement 3 (C3) in the lower epidermal keratinocytes (Fig 2). Indirect immunofluorescence (IIF) and enzyme-linked immunosorbent assay (ELISA) or desmoglein (Dsg) 1 and 3 were within normal limits and antinuclear antibodies (ANAs) were negative. The patient showed improvement with tacrolimus 0.1% ointment.

Pemphigus foliaceus (PF) is an acquired autoimmune blistering disorder in which the immune system creates IgG antibodies that attack the intercellular adhesion glycoprotein Dsg 1, resulting in subcorneal blisters and acantholysis.^1^^,^^2^ Worldwide incidence and prevalence of PF is low. Localized PF is a rarely reported entity in the literature, with even fewer reported cases isolated to the nose.^1^, ^2^, ^3^ In current literature, cases of PF localized to the nose have remained confined, whereas others progressed beyond the nose.^2^^,^^3^ Localized pemphigus seems to favor seborrheic areas of the face, specifically the nose, though the reason is not completely clear.^2^^,^^3^ Previously reported cases have demonstrated various combinations of Dsg 1 and Dsg 3 antibodies, including negative Dsg 1 antibodies with positive Dsg 3 antibodies, as well as initially positive Dsg 1 antibodies with negative Dsg 3 antibodies later progressing to positive antibodies for both Dsg 3 and Dsg 1.^2^ It has been suggested that PF in sun-exposed areas may be attributed to the increased expression of Dsg 1 antigen in the face or increased exposure to ultraviolet light, as it may induce the binding of anti-Dsg 1 to the epidermis.^2^^,^^3^ Literature describing treatments of localized nasal PF includes variations of systemic corticosteroids, topical corticosteroids, calcineurin inhibitors, dapsone, and other immunosuppressive agents.^3^^,^^4^

Seronegativity in this case is suspected to be due to the localized nature of PF. Although ELISA and IIF are sensitive in detecting circulating anti-Dsg autoantibodies in widespread pemphigus foliaceus (PF), localized PF may involve antibody levels below detection thresholds or antibody sequestration within affected skin. Therefore, negative ELISA and IIF do not exclude PF, and biopsy with DIF becomes critical for diagnosis of localized PF. Interestingly, DIF in this patient showed intercellular IgG deposition confined to the lower half of the epidermis. In classic PF, staining is typically more prominent in the upper epidermis, correlating with Dsg 1 distribution.^5^ Further studies are needed to determine whether this staining pattern is distinct to localized presentations or reflects temporal disease evolution.

In conclusion, localized involvement of the nose is a rare presentation of pemphigus foliaceus. It seems to follow a more benign course and may be treated topically with topical corticosteroids or calcineurin inhibitors with escalation to systemic therapies if needed.^2^^,^^4^ Additional studies are needed to understand the pathogenesis of localized PF and what determines its progression to more extensive disease to provide optimal treatment.

## Conflicts of interest

Brittney Schultz, MD: Argenx—Advisory Board (honoraria), AstraZeneca—Sub-investigator, UCB—Investigator, Regeneron/Sanofi—Advisory Board (honoraria).
